# Sedation, sleep-promotion, and non-verbal and verbal communication techniques in critically ill intubated or tracheostomized patients: results of a survey

**DOI:** 10.1186/s12871-022-01887-z

**Published:** 2022-12-12

**Authors:** Christian Waydhas, Teresa Deffner, Robert Gaschler, David Häske, Uwe Hamsen, Frank Herbstreit, Anke Hierundar, Oliver Kumpf, Georg Rohe, Aileen Spiekermann, Sonja Vonderhagen, Reiner M. Waeschle, Reimer Riessen

**Affiliations:** 1 Ruhr-Universität-Bochum, Universitätsstrasse 150, 44801 Bochum, Germany; 2 Klinik Und Poliklinik Für Chirurgie, Berufsgenossenschaftliches Universitätsklinikum Bergmannsheil Bochum, Bürkle-de-La-Camp-Platz 1, 44789 Bochum, Germany; 3 Present Address: Klinik Für Unfallchirurgie, Universitätsklinikum, Universitätsmedizin Essen, Hufelandstr. 55, 45147 Essen, Germany; 4 Klinik Für Anästhesiologie Und Intensivmedizin, Universitätsklinikum Jena, Bachstrasse 18, 07743 Jena, Germany; 5 Fakultät Für Psychologie, Lehrgebiet Allgemeine Psychologie: Lernen, Motivation, Emotion, FernUniversität in Hagen, Universitätsstrasse 33, 58084 Hagen, Germany; 6 Center for Public Health and Health Services Research, University Hospital Tübingen, Osianderstraße 5, 72076 Tübingen, Germany; 7 Klinik Für Anästhesiologie Und Intensivmedizin, Universitätsklinikum, Universitätsmedizin Essen, Hufelandstr. 55, 45147 Essen, Germany; 8 Klinik Für Anästhesiologie Und Intensivtherapie, Universitätsmedizin Rostock, Schillingallee 35, 18057 Rostock, Germany; 9 Klinik Für Anästhesiologie Mit Schwerpunkt Operative Intensivmedizin, Campus Charité Mitte Und Campus Virchow-Klinikum, Charité – Universitätsmedizin Berlin, corporate member of Freie Universität Berlin, Humboldt-Universität Zu Berlin, and Berlin Institute of Health, Charitéplatz 1, 10117 Berlin, Germany; 10 University Clinic for Anaesthesiology / Intensive Care Medicine / Emergency Medicine / Pain Medicine, Klinikum Oldenburg, Medical Campus of the University Oldenburg), Rahel Straus - Str. 10, 26133 Oldenburg, Germany; 11 Klinik Für Anästhesiologie, Universitätsmedizin Göttingen, Robert-Koch-Str. 40, 37099 Göttingen, Germany; 12 Department Für Innere Medizin, Universitätsklinikum Tübingen, Otfried-Müller-Str. 10, 72076 Tübingen, Germany

**Keywords:** Sedation, Sleep management, Communication, Intensive care, Mechanical ventilation

## Abstract

**Background:**

The aim of this survey was to describe, on a patient basis, the current practice of sedation, pharmacologic and non-pharmacologic measures to promote sleep and facilitation of communication in critically ill patients oro-tracheally intubated or tracheostomized.

**Methods:**

Cross-sectional online-survey evaluating sedation, sleep management and communication in oro-tracheally intubated (IP) or tracheostomized (TP) patients in intensive care units on a single point.

**Results:**

Eighty-one intensive care units including 447 patients (IP: *n* = 320, TP: *n* = 127) participated. A score of ≤ -2 on the Richmond Agitation Sedation Scale (RASS) was prevalent in 58.2% (IP 70.7% vs. TP 26.8%). RASS -1/0 was present in 32.2% (IP 25.9% vs. TP 55.1%) of subjects. Propofol and alpha-2-agonist were the predominant sedatives used while benzodiazepines were applied in only 12.1% of patients. For sleep management, ear plugs and sleeping masks were rarely used (< 7%). In half of the participating intensive care units a technique for phonation was used in the tracheostomized patients.

**Conclusions:**

The overall rate of moderate and deep sedation appears high, particularly in oro-tracheally intubated patients. There is no uniform sleep management and ear plugs and sleeping masks are only rarely applied. The application of phonation techniques in tracheostomized patients during assisted breathing is low. More efforts should be directed towards improved guideline implementation. The enhancement of sleep promotion and communication techniques in non-verbal critically ill patients may be a focus of future guideline development.

**Supplementary Information:**

The online version contains supplementary material available at 10.1186/s12871-022-01887-z.

## Background

The management of sedation appears to be associated with duration of ventilation, morbidity, and length of stay of mechanically ventilated patients in the intensive care unit (ICU) [1–3]. National and international guidelines [4, 5] assist medical teams in optimizing sedation protocols and bedside management. The general strategy is to reduce the amount of sedative medication as soon as possible, achieving light sedation, by using regular sedation breaks or straight forward protocols reducing the rate of delirium and the length of invasive ventilation [4, 5]. However, it has been shown previously, that the guideline recommendations are often not consistently implemented [6–12]. Therefore, sedation management has been one of the main topics for quality improvement programs in the ICU. Sedation management is one of the 10 national quality indicators in Germany [13, 14] and the consistent use of a weaning protocol including sedation management is relevant for reimbursement in the German DRG-system [15]. Only few indications for deep sedation in mechanically ventilated patients remain, such as increased intracranial pressure, status epilepticus, severe ARDS, acute shock and a few others [4]. The ideal state of consciousness is thought to be in the range from -1 to 0 on the Richmond Agitation Sedation Scale (RASS) or equivalent [4, 5, 16]. Some patients awake on mechanical ventilation might suffer from sleep deprivation and psychological strain, intensified by the inability to communicate verbally with an artificial airway in place [17, 18]. Many previous surveys have investigated the concepts and organization of sedation management in ICUs on an institutional level [6, 8, 12, 19–23], but only few have focused on individual patient management [3, 7, 24].

The aim of this survey was to describe, on a patient basis, (a) the present practice of sedation, (b) pharmacologic and non-pharmacologic measures to promote sleep and (c) facilitation of communication in critically ill patients with artificial airways in German ICUs. A secondary objective was to specify potential differences between oro-tracheally intubated and tracheostomized patients.

## Methods

We conducted a cross-sectional electronic survey following the CROSS-checklist [25]. The research questions were strictly focused on sedation, sleep promotion and communication of intubated or tracheostomized patients treated in the intensive care unit.

### Questionnaire

The survey questions were developed by members of the section “Quality in Intensive Care Medicine” of the German Interdisciplinary Association of Critical Care and Emergency Medicine (DIVI) and reviewed by other specialists. The group consisted of intensivists, intensive care psychologists, public health specialists and specialists in the development of electronic survey forms. The questions were built on a review of the current literature and guidelines, including the most recent update of the German S3-guideline “Analgesia, Sedation and Management of Delirium in Intensive Care” [4, 5]. The survey questions were programmed with the commercial software “Umfrage Online” in German language (https://www.umfrageonline.com). The survey was then tested in two rounds of pilot trials in 5 ICUs and further adapted for clarity, consistency and face validity and then approved by the executive committee of the DIVI. The survey included 23 questions and required about 15 to 30 min to complete. The items consisted mostly of closed questions (single and multiple-choice questions) and a few open questions. The translation of the survey questions into English can be found in the electronic supplement 1).

### Survey

The survey was designed to collect single-point data. The status of every intubated or tracheostomized Patient in the ICU on the morning of the index day or, in some questions, referring to the preceding 12 or 24 h was assessed. The survey was conducted from February 7, 2022 to March 31, 2022. The index day could be freely chosen within this period. All questions had to be answered to complete the survey.

The survey was sent to all German intensive care units included in the society’s (DIVI) database. Overall, 1281 hospitals and their ICUs were contacted. These included medical, surgical, specialized as well as mixed medical and surgical ICUs. There was only one recipient per ICU so that multiple answers from one ICU were avoided.

### Data collection and analysis

The raw data were downloaded and exported to an excel file (Microsoft® Excel for Mac, version 2019). Statistical analysis was performed with the Chi-square test. Significance was assumed with a *p* < 0.01.

The survey was completely anonymous with respect both to patients and to participating physicians / intensive care units. Therefore, no ethical approval was required. Consent of the participants was assumed by completing the survey.

## Results

**Fig. 1:**
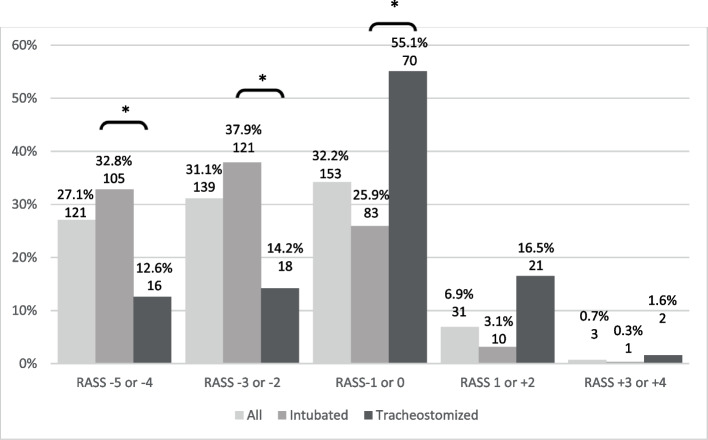
Distribution of the level of the Richmond Agitation Sedation Scale (RASS) on the morning of the index day (percent within group, number of patients). * *p* < 0.001

Eighty-one intensive care units responded with complete data sets including 447 oro-tracheally intubated or tracheostomized patients, averaging 5.5 patients per ICU. There were 320 oro-tracheally intubated and 127 tracheostomized patients. 62 of the 81 ICUs reported treating tracheostomized critically ill patients on the index day. The distribution of the level of sedation / agitation is shown in Fig. 1. The group with RASS -1/0 (awake and calm) included 32.2% of patients. However, the total of patients with RASS ≤ -2 (sum of patients with moderate (RASS -2/-3) and deep (RASS -4/-5) sedation was prevalent in 58.2% of patients. Depth of sedation differed significantly between intubated and tracheostomized patients. About 55% of the tracheostomized patients were in the awake group (RASS-1/0), compared to 25.9% of the intubated patients. The rate of moderate or deep sedation was 70.7% vs. 26.8% for the intubated and tracheostomized patients, respectively.

Delirium (according to CAM-ICU or Nu-DESC ≥ 2 points or to other validated delirium scores) within the preceding 12 h was present in 19.9% (*n* = 89) of patients, being similar in the intubated and tracheostomized group (19.7% vs. 20.5%).

**Fig. 2:**
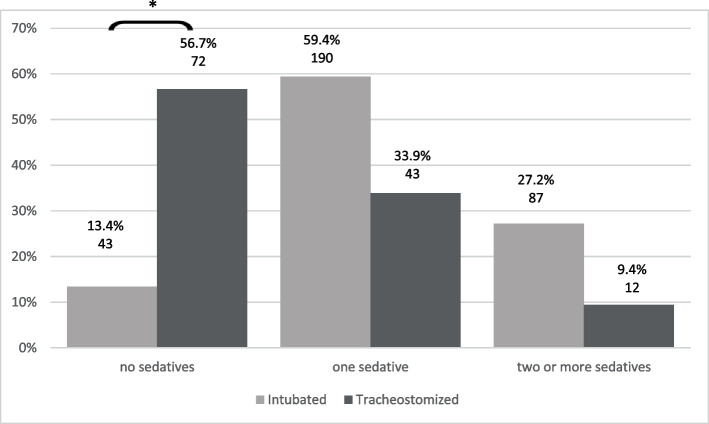
Use of sedative medication (percent within group IP and TP, number of patients). * *p* < 0.001

In tracheostomized patients the use of sedatives was significantly lower compared to the intubated group (Fig. 2). More than half of the tracheostomized subjects (56.7%) did not receive any sedatives, while only 13.4% of the intubated patients were sedative-free. Similarly, the type of sedative used differed considerably between the two groups (Table 1). In intubated patients propofol was the most widely used sedative, whereas dexmedetomidine and clonidine predominated in the tracheostomized subjects. Volatile sedatives were only used in a minority of about 5% of patients.

**Table 1:** Type of sedative drug used. Number of patients with percentage in parentheses

	All patients (*n* = 447)	Oro-tracheally intubated patients (*n* = 320)	Tracheostomized patients (*n* = 127)
Benzodiazepines	54 (12.1%)	47 (12.3%)	7 ( 8.2%)
Propofol	187 (41.8%)	173 (45.3%)	14 (16.5%)
Volatile sedatives	21 (4.7%)	19 ( 5.0%)	2 ( 2.4%)
Dexmedetomidine	89 (19.9%)	64 (16.8%)	25 (29.4%)
Clonidine	81 (18.1%)	52 (13.6%)	29 (34.1%)
Other	35 (7.8%)	27 ( 7.0%)	8 ( 9.4%)

A wide variety of medications was used to promote sleep (Table 2). In general, more sleeping medication was administered in tracheostomized patients. Non-pharmacological interventions promoting sleeping, such as ear plugs or sleeping masks were only rarely used in both groups.

**Table 2:** Pharmacological^a^ and non-pharmacological support of sleeping

	Oro-tracheally intubated patients (*n* = 320)	Tracheostomized patients (*n* = 127)
Zopiclone	0	3 (2.4%)
Melatonin	33 (10.3%)	30 (23.6%)
Benzodiazepine	13 (4.1%)	6 (4.7%)
Clonidine	40 (12.5%)	20 (15.7%)
Melperone	28 (8.7%)	19 (15.0%)
Neuroleptic agents	28 (8.7%)	25 (19.7%)
Propofol	20 (6.2%)	6 (4.7%)
Other drugs	7 (2.2%)	7 (5.5%)
Ear plugs	22 (6.9%)	6 (4.7%)
Sleeping masks	5 (1,6%)	1 (0.8%)

^a^The medication was not used for sedation, but explicitly for sleep promotion

With respect to the type, frequency, and ability to communicate significant differences between intubated and tracheostomized patients were observed. While communication via winking/nodding and the use of a letter board/writing was possible in 38.1% (*n* = 113) and 8.1% (*n* = 26) in the intubated group, respectively, tracheostomized patients communicated via winking/nodding in 70.1% (*n* = 89) and used a letter board/writing in 26.8% (*n* = 34) of cases.

In the presence of a tracheostomy tube phonation and verbal communication is potentially possible. This was the case in 37.8% of patients on the index day. However, phonation was administered in only the half of ICUs (33 of 62). When phonation was used, it was employed by nurses in 100% of subjects, by physicians (in 93.9%), by therapists (in 75.8%) and less often by family members (in 63.6%).

Finally, the participating ICUs were asked how they try to allow for phonation in general (Table 3). Speaking with a standard tracheal cannula during spontaneous breathing with a deflated cuff and a speaking valve was by far the technique used most, as compared to the use of fenestrated tracheal tubes. Speaking during assisted breathing was achieved in only a quarter of the participating ICUs. An electrolarynx and other techniques were used rarely.

**Table 3:** Type of phonation procedure in tracheostomized patients in the ICU in general

Number of ICUs	62
No phonation procedures in use	1 (1.6%)
Spontaneous breathing, cuff deflated, speaking valve	50 (80.6%)
Spontaneous breathing, tracheal tube with phonation opening	17 (27.4%)
Assisted breathing, cuff deflated	12 (19.4%)
Assisted breathing, tracheal tube with phonation opening	7 (11.3%)
Electrolarynx	1 (1.6%)
Using the subglottic suctioning opening	2 (3.2%)

## Discussion

In our survey more than 58% of all patients with an artificial airway and more than 70% of the subgroup of patients with an oro-tracheal tube were kept in moderate or deep sedation (RASS ≤ -2) in our survey. This is in a similar range as the 74% of mechanically ventilated patients with a RASS of < -2 in a European study [10]. In another recent study patients with assisted breathing were reported to be deeply sedated (RASS ≤ -3) on more than 75% of all days throughout their ICU-stay [3]. Two Spanish studies [21, 24] reported 22.6% of their patients to be in deep (RASS ≤ -4) and 41.3% in moderate or deep (RASS ≤ -2) sedation, which is somewhat less than our overall rate of deep (27.1%) and moderate or deep sedation (58.2%), respectively. Correspondingly 45.7% of their patients were reported to be in the RASS range of -1 to 0 [21]. However, they have included intubated as well as non-intubated subjects, so more awake and less sedated patients would be expected, compared to the group of patients with artificial airway. Although many guidelines advocate light sedation [4, 5], the real-life fraction of deep or moderately deep sedated patients is still considerably high throughout many European and non-European intensive care units. Interestingly, a recent meta-analysis and systematic review showed an improvement on intensive care mortality and reduction in the duration of ventilation only in cohort studies, but not in randomized controlled trials [26]. At the same time, they reported of no difference in adverse events (delirium, self-extubation, re-intubation) [26]. However, some studies indicate that lighter sedation may result in more stressful memories [27] and up to 42.6% of mechanically ventilated patients would have desired more sedation [18]. On the other hand deep sedation does not protect patients from feelings of anxiety, but hinders their ability to communicate [28].

Not surprisingly, the fraction of awake patients was significantly higher and the rate of deep sedation lower, in tracheostomized patients. This has been reported before [29].

Although tracheostomy itself is not recommended as a means for reducing sedation, in a randomized trial significantly less intravenous sedation and less periods of deep sedation were observed in the early tracheostomy group [30]. Tracheostomy tubes appear to be better tolerated and may result in a different sedation practice, but for specific patients with respiratory insufficiency it has been shown that tracheostomy per se did not lead to a higher RASS level [31].

Only 13.4% of oro-tracheally intubated patients but 56.7% of the tracheostomized subjects did not receive any continuous intravenous sedatives. In our study propofol was by far the predominate sedative agent used (41.8%) while benzodiazepines were only applied in a small minority. This finding is in conflict with the results of most other studies where benzodiazepines were the sedatives used most often [3] or applied with the same frequency as propofol [6, 21, 22]. In the past, benzodiazepines have been the first choice for sedation, also in German ICUs, for sedation requirements of longer than 24 h [11]. However, a trend towards a reduced use of midazolam has been observed in the years thereafter [32]. Our data indicate that this development has continued, and benzodiazepines are no longer widely used. It may be assumed that the strong recommendations of major guidelines during the last decade [4, 5, 33, 34] that advised against the use of benzodiazepines due to an increased risk of developing delirium are now commonly accepted and are implemented in the daily practice. Future efforts should concentrate on adopting new concepts and methods to establish lighter sedation goals in daily practice [35].

Alpha-2-agonists were broadly used in our survey in a similar range as has been reported in other studies (10.1% to 65.1%) [6, 22]. They were the predominant sedatives used in our group of tracheostomized patients. It is of note that dexmedetomidine and clonidine were used with similar frequencies, while in the international literature only dexmedetomidine appears to be applied, although little data on the comparative effects of both drugs are available [36].

Volatile sedatives were rarely used in our survey, maybe because the approval of the European authorities for long-term sedation in critically ill patients has been given only recently. In a survey of French ICUs 21% of ICU directors declared that they routinely use volatile sedatives [37]. However, there was no indication of how often volatile sedatives were applied.

While sleep deprivation is a common problem in ICU patients [38], recent guidelines give no clear recommendations which specific methods (apart from implementing a noise and light reduction concept) or agents should be used [4, 5]. This lack of clear guidance is reflected by the large variety of sleeping drugs used in our patients without any clear favorite in the oro-tracheally intubated patients. In the tracheostomized subjects, melatonin and neuroleptic agents were preferred each in about 20% of patients. Other investigators have reported a very variable use of sleep medication in 13% to 58% of ICU-patients [12, 22, 39].

Benzodiazepines appear to be the preferred agent [12], but also dexmedetomidine and propofol are used [22]. In contrast, benzodiazepines (and propofol) were used very rarely in our study.

With more intubated or tracheostomized patients being more awake the requirement for communication increases. However, 61.9% of the intubated patients were not even able to communicate by winking or nodding. In one study 53.9% of mechanically ventilated patients met basic communication criteria [40]. The medical staff reported difficulty in communicating with patients during 35% of ICU days [41]. Mechanically ventilated patients reported “being afraid”, “feeling supervised”, “failing to communicate”, and “experiencing difficulties in breathing” with a frequency of 66.6% [42]. Being dependent on health professionals, without being able to communicate and not being understood, causes experiences of misunderstanding, loss of control, dependency, anxiety, fear, and loneliness. [43, 44]. The inability to communicate was significantly associated with a loss of control and helplessness and impacted negatively on satisfaction with care [45]. One typical quotation of a patient was "not being able to talk was horrid".

Augmentative and alternative communication (AAC) can improve patient satisfaction [46]. Simple means of communication such as winking/nodding were regularly used in intubated patients whereas the use of a letter board or writing was possible in less than 10% of patients. These techniques allowed for communication twice or three times as often in tracheostomized patients. We are not aware of other studies reporting the frequency of such basic communication.

In tracheostomized patients, several techniques allow for speaking with the tracheostomy tube in place. On the index day phonation was practiced in more than one third of tracheostomized patients, but in only half of the ICUs that cared for such patients. In general, the predominantly used phonation procedures included the use of cuff deflation and a speaking valve with either a standard cannula or a fenestrated tracheostomy tube in patients breathing spontaneously without mechanical assistance. Far less ICUs applied these techniques to patients with assisted breathing. It has been shown, however, that cuff deflation during mechanical ventilation results in a significantly earlier phonation than cuff deflation only during self-ventilation (7 vs. 18 days) [47]. A recently described technique of vocalizing by administering air flow through the subglottic suctioning port [48] was used in two ICUs.

Our study has several limitations. The low number of participating ICUs (*n* = 81) and the low response rate may preclude conclusions representative for all ICUs. However, no selection bias on the side of the authors is present. The cause for the low response rate is not clear but may be due to a high workload on the side of the potential participants, particularly in the wake of the COVID pandemic. Also, the relatively high workload of filling out all questions for each patient and then aggregating the data for the input into the survey form may have limited the participation rate. On the other hand, we believe that the data from those who put in the effort are quite reliable. They may even be more reliable compared to surveys that ask about concepts and practices in a general way, when some bias toward answers that reflect an eligible opinion may be present. It cannot be ruled out that the low response rate might reflect an exclusion of more complex patients who needed higher efforts in clinical treatment or a low response by ICU with high volume of complex patients. However, the number of 5.5 intubated or tracheostomized patients per ICU included in our study appears to be well in the range of ventilated patients in German ICUs on average (electronic supplement 2), so that a major bias appears not to be very likely.

Intubated and tracheostomized patients are usually not comparable in terms of timing and thus of relative need for sedation, of adaptation to ventilation, or of communication skills. Therefore, we reported the results separately for both groups. As this is a survey, no power analysis or estimation of relative sample size was performed.

We cannot connect sedative use neither to patient-specific characteristics such as age, diagnoses (such as the fraction of unstable patients with shock, ARDS, acute severe neurologic disorders) or RASS-groups nor to the type of ICU (medical, surgical, mixed). Furthermore, we did not gather information about pain management which may interact with sedation requirements. However, our aim was to describe the present practice of sedation, sleep promotion and communication of intubated and tracheostomized patients in the ICU. A detailed analysis of the actual sedation management in different patient groups and an exploration of possible causes for the deeper than expected sedation would require another study with a much more extensive data acquisition. Despite these limitations our study provides insights into the present actual sedation management of many ICUs and adds new information about the practice of sleep management and applied communication with oro-tracheally intubated and tracheostomized patients.

## Conclusions

Our study shows that the number of oro-tracheally intubated patients with a RASS ≤ -2 is high. Only a quarter of those patients achieve an awake and calm state as targeted by current guidelines. In contrast, the majority of tracheostomized patients is in a RASS-range of -1/0 receiving significantly less sedatives. Benzodiazepines are only rarely used in German ICUs. There is no predominant concept of sleep management and non-pharmacologic interventions (ear plugs, sleeping masks) are rarely used. Although techniques that allow phonation in tracheostomized patients are well known, the application rate appears relatively low, particularly in patients with assisted breathing. Future studies are needed to understand whether recent new guidelines and the still rising interest in sedation management and communication techniques will change the behavior in patients receiving ventilator support.

## Supplementary Information

**Additional file 1.**

**Additional file 2.**

## Data Availability

The datasets used and/or analysed during the current study are available from the corresponding author on reasonable request.

## Abbreviations

AAC: Augmentative and Alternative Communication
ARDS: Adult Respiratory Distress Syndrome
CROSS: Checklist for Reporting Of Survey Studies
DIVI: German Interdisciplinary Association of Critical Care and Emergency Medicine
DRG: Diagnosis-Related Group
ICU: Intensive Care Unit
RASS: Richmond Agitation Sedation Scale
