# Supplementary material for: Sedation, sleep-promotion, and non-verbal and verbal communication techniques in critically ill intubated or tracheostomized patients: results of a survey
Source: BMC Anesthesiol. 2022 Dec 12;22:384. doi: 10.1186/s12871-022-01887-z (PMC9743767; doi:10.1186/s12871-022-01887-z)
Supplement: Supplementary file 1 — Additional file 1. [file 12871_2022_1887_MOESM1_ESM.docx]

**Supplemental Material:**

**English translation of the survey**

**Sedation and communication in intubated and tracheostomized patients**

***Page 1***

Dear colleagues,

Yet again a survey from the DIVI, you might think. Nevertheless, we ask you to take a quick look at the questions and take part in the survey. Recently, an updated version of the DAS guideline on *Sedation, Analgesia and Delirium Management in Intensive Care* has been published. It has been organized under the leadership of the DGAI and the DIVI and many other professional societies participated. Key elements of the guideline include: Sedation of intensive care patients and communication with intubated and tracheostomized patients.

With this survey we hope to obtain representative information regarding the current state of sedation of intubated and tracheostomized patients, the ways in which non-verbal patients can communicate with their environment and receive information about sleep-promoting measures. The information you provide will be used to identify areas where there may be a need for further research and and/or quality enhancing measures and to assess potential effects of the updated guideline on future clinical practice. This survey will take about 15-30 minutes to complete.

Your information will be anonymized completely and immediately. Please provide details as accurately as possible. The results will be presented at the DIVI Congress and will be published freely available to you. The Quality Section of the DIVI, on behalf of the entire DIVI Executive Board, would like to invite you to participate in order to be able to make a statement that is as representative as possible on important topics concerning intensive care medicine.

The data are to be collected on one day during morning rounds. We recommend printing out the questions in the PDF file you have received with the invitation e-mail and take it with you as a paper aid for the collection of data during your round and then enter the data into the online survey.

If you have any questions, please contact christian.waydhas@uni-due.de

Please press NEXT now.

***Page 2***

Are there any oro-/naso-tracheally intubated patients in your intensive care unit **this morning**?

No

Yes. Please indicate the number of patients!

***Page 3***

***The following questions deal exclusively with oro-tracheally (or naso-tracheally) intubated patients!***

***Applies only if patients are oro-/naso-tracheally intubated:***

**This morning**, how many of the oro/naso-tracheally intubated patients had a RASS (or equivalent) of:

RASS -5 or -4 (no response to loud voice OR response only to physical stimulus or no response at all)

RASS -3 or -2 (awakens shortly (less than 10 seconds) with eye contact on verbal stimulus OR any movement (but without Eye contact) with verbal stimulus)

RASS -1 or 0 (quiet and alert or not fully alert OR sleepy but persistently awake (greater than 10 seconds), with eye contact, on verbal stimulus)

RASS +1 or +2 (fearful but no aggressive or lively movement OR frequent untargeted movement, breathes against the respirator)

RASS +3 or +4 (draws or removes tubes or catheters, or shows aggressive behavior towards personnel OR manifestly aggressive or violent, poses immediate danger to staff)

***Page 4***

***Applies only if patients are oro-/naso-tracheally intubated:***

How many of the oro-/naso-tracheally intubated patients experienced delirium **in the past 12 hours**?

***Page 5***

***Applies only if patients are oro-/naso-tracheally intubated:***

How many of the oro-naso-tracheally intubated patients received a continuous infusion of sedatives (benzodiazepine, propofol, volatile sedative, dexmedetomidine, clonidine, other sedative) **this morning**? (Multiple selections possible.)

No continuous infusion of sedatives

Continuous infusion with one sedative

Continuous infusion with 2 or more sedatives at the same time

***Page 6***

***Applies only if patients are oro-/naso-tracheally intubated:***

How many of the oro-naso-tracheally intubated patients received a continuous infusion with one of
the following sedatives **this morning**? (In case of concomitant administration of 2 or more sedatives,
count each substance. Multiple selections possible.)

Benzodiazepine

Propofol

Volatile sedative

Dexmedetomidine

Clonidine

Other

***Page 7***

***Applies only if patients are oro-/naso-tracheally intubated:***

How many of the oro-/naso-tracheally intubated patients have answered yes/no questions by nodding / shaking their head or by winking or other movements **this morning**?

***Page 8***

***Applies only if patients are oro-/naso-tracheally intubated:***

How many of the oro/naso-tracheally intubated patients have communicated via response panels or through written messages **this morning**?

***Page 9***

***Applies only if patients are oro-/naso-tracheally intubated:***

How many of the oro/naso-tracheally intubated patients received one of the following sleeping pills / neuroleptics **last night**? (Multiple selections possible.)

Zopiklon

Melatonin (or analogue)

Benzodiazepine

Clonidine

Melperon

Other neuroleptic agent (haloperidol, quetiapine, promethazine, etc. )

Propofol (explicitly as sleeping remedy only at night)

Other

***Page 10***

***Applies only if patients are oro-/naso-tracheally intubated:***

How many of the oro-/naso-tracheally intubated patients received earplugs **last night**?

***Page 11***

***Applies only if patients are oro-/naso-tracheally intubated:***

How many of the oro-/naso-tracheally intubated patients received sleep masks **last night**?

***Page 12***

***The following questions deal exclusively with tracheostomized patients!***

Are there any tracheostomized patients in your intensive care unit **this morning**?

No

Yes. Please indicate the number of patients!

***Page 13:***

***Applies only if patients are tracheostomized:***

**This morning**, how many of the tracheostomized patients had a RASS (or equivalent) of:

RASS -5 or -4 (no response to loud voice OR response only to physical stimulus or no response at all)

RASS -3 or -2 (awakens shortly (less than 10 seconds) with eye contact on verbal stimulus OR any movement (but without eye contact) with verbal stimulus

RASS -1 or 0 (quiet and alert or not fully alert OR sleepy but persistently awake (greater than 10 seconds), with eye contact, on verbal stimulus)

RASS +1 or +2 (fearful but no aggressive or lively movement OR frequent untargeted movement, breathes against the respirator)

RASS +3 or +4 (draws or removes tubes or catheters, or shows aggressive behavior towards personnel OR manifestly aggressive or violent, poses immediate danger to staff)

***Page 14***

***Applies only if patients are tracheostomized:***

How many of the tracheostomized patients experienced delirium **in the past 12 hours**?

***Page 15***

***Applies only if patients are tracheostomized:***

How many of the tracheostomized patients received a continuous infusion of sedatives (benzodiazepine, propofol, volatile sedative, dexmedetomidine, clonidine or another sedative) **this morning**? (Multiple selections possible.)

No continuous infusion of sedatives

Permanent infusion with a sedative

Continuous infusion with 2 or more sedatives at the same time

***Page 16***

***Applies only if patients are tracheostomized:***

How many of the tracheostomized patients had received a continuous infusion of one of
the following sedatives **this morning**? (In case of concomitant administration of 2 or more sedatives, count each substance. Multiple selections possible)

Benzodiazepine

Propofol

Volatile sedative

Dexmedetomidine

Clonidine

Other

***Page 17***

***Applies only if patients are tracheostomized:***

How many of the tracheostomized patients have answered yes/no questions by nodding / shaking their head or by winking or other movements **this morning**?

***Page 18***

***Applies only if patients are tracheostomized:***

How many of the tracheostomized patients have communicated via response panels or through written messages **this morning**?

***Page 19***

***Applies only if patients are tracheostomized:***
How many of the tracheostomized patients received one of the following sleeping pills / neuroleptics **last night**? (Multiple selections possible.)

Zopiklon

Melatonin (or analogue)

Benzodiazepine

Clonidine

Melperon

Other neuroleptic agent (haloperidol, quetiapine, promethazine, etc. )

Propofol (explicitly as sleeping remedy only at night)

Other

***Page 20***

***Applies only if patients are tracheostomized:***

How many of the tracheostomized patients received earplugs **last night**?

***Page 21***

Applies only if patient is tracheostomized:
How many of the tracheostomized patients received sleep masks **last night**?

***Page 22***

***Applies only if patients are tracheostomized:***

How many of the tracheostomized patients have had a device in place that allows phonation with a tracheal cannula and communicated **in the past 24 hours**?

None

Otherwise, please indicate the number of patients!

***Page 23***

***Applies only if patients are tracheostomized:***
Who has communicated with these patients using these talking devices, or phonation aids **in the past 24 hours**? (Multiple selections possible.)

Nurse

Physiotherapist

Physician

Family member /visitor

Other

***Page 24***

***Applies only if patients are tracheostomized:***

What communication devices and aids are used in general in tracheostomized patients on your unit **in general**? (Multiple selections possible.)

Unblocked cannula with talking valve for spontaneous breathing

Unblocked channels under assisted ventilation

Cannula with phonation opening for spontaneous breathing

Cannula with phonation opening under assisted breathing

Electrolarynx

Other (please specify)

***Page 25***

You have completed the survey. If you would like to make any corrections, you can go back.

Thank you very much for taking the time to participate.
Quality Section of the DIVI

The survey is now complete. This window can now be closed.
