# Supplementary material for: Sedation, sleep-promotion, and non-verbal and verbal communication techniques in critically ill intubated or tracheostomized patients: results of a survey
Source: BMC Anesthesiol. 2022 Dec 12;22:384. doi: 10.1186/s12871-022-01887-z (PMC9743767; doi:10.1186/s12871-022-01887-z)
Supplement: Supplementary file 2 — Additional file 2. [file 12871_2022_1887_MOESM2_ESM.docx]

Manuscript „Sedation, sleep-promotion, and non-verbal and verbal communication techniques in critically ill intubated or tracheostomized patients: results of a survey

Waydhas et al.

Electronic Supplement 2

In German ICUs, the rate of invasively ventilated patients has been reported to vary between 13% and 59.8% [1-3]. Accordingly, we assume that the actual rate of ventilated patients is somewhere between 25% and 50%. Taking the average of 5.5 ventilated patients per ICU in our study, it would result in an average ICU size between 11 and 22 beds.

In the German “DIVI-Intensivregister” (DIVI intensive care registry) 21,730 ICU beds from 1323 ICUs were included (on March 30,2022, end of our survey) [4]. This would result in an average size of 16,3 beds per ICU.

Thus, the average number of beds per ICU in Germany corresponds well with the estimated number of ICU beds in the ICUs participating in our survey.

1. Kaier K, Heister T, Wolff J, Wolkewitz M. Mechanical ventilation and the daily cost of ICU care. BMC Health Serv Res. 2020;20:267.

2. Folsch C, Kofahl N, Waydhas C, Stiletto R. [Cross sectional study of structural quality of German intensive care units. A reevaluation of the DIVI register]. Med Klin Intensivmed Notfmed. 2013;108:497-506.

3. Bingold TM, Lefering R, Zacharowski K, Waydhas C, Scheller B. [Eleven years of core data set in intensive care medicine. Severity of disease and workload are increasing]. Anaesthesist. 2014;63:942-50.

4. Deutsche Interdisziplinare Vereinigung fur Intensiv- und Notfallmedizin e V. DIVI-Intensivregister. 2022. (<https://www.intensivregister.de/#/aktuelle-lage/zeitreihen>). . Accessed 1.8. 2022.
